# Supplementary material for: Unconscious Drivers of Consumer Behavior: An Examination of the Effect of Nature–Nurture Interactions on Product Desire
Source: Behav Sci (Basel). 2024 Sep 7;14(9):789. doi: 10.3390/bs14090789 (PMC11428589; doi:10.3390/bs14090789)
Supplement: Supplementary file 1 [file behavsci-14-00789-s001.zip › behavsci-3114224-supplementary.pdf]

Scenario Stories Used in Experiment

<u>Social Support: Safe condition</u>

You walk down the carpeted hall and turn the doorknob, happy to find that it's not locked because your partner must be home. As you push open the door, savory spices waft from the kitchen—your partner must be cooking. Then suddenly you are gaping at a room full of beautiful smiling faces and greeted by a joyous shout: SURPRISE! And it really IS a surprise. Your jaw drops and you let out a breath as the blood rushes to your face. You feel your smile widening into a huge grin. And they're all there. You knew your partner was getting a lot of phone calls at home lately, but now you know why; they've been setting this up for weeks. Your mum and dad are there, each with an arm round your partner, who can't stop laughing, right there in the middle of everyone. Your two sweet sisters and your brother, who's wearing a little cone birthday party hat, blowing one of those wheezy stretch-whistles and making one of his usual goofy faces. The whole family has gathered from across three provinces to be here for your birthday, and you never had a clue. You suddenly notice your best friend from back home, who must have driven up here with your parents. A tear runs down your old friend's cheek, next to that gigantic smile you remember so well, and have missed so much lately, in spite of all your Facebook conversations. Your own eyes start to moisten and you bite your lip a little to keep the happy-tears back. Two of your closest friends are there from work, and three of your neighbors, including the sweet, hard-of-hearing old lady from down the hall who always brings you cookies. They all seem to have gotten to know one another before you even got here. It's like your family has not only suddenly appeared from out of the blue, but has expanded instantly to include your favorite people from here in the city.

You remember saying goodbye to some of these same folks a few years ago and coming to a city where you didn't know a soul and how lonely that was at first. That feeling has been gone for a long time now, and tonight you feel connected to everyone here, and connected to this city, your home now. And you feel surrounded by love.

Pause for a moment and think about the story you just read. How would you feel if this really was your situation?

<u>Social Support: Harsh condition</u>

You trudge down the hallway, fish for your key, and your heart sinks. A few months ago that door would have been unlocked, and your partner would be waiting for you. Tonight as you push open the door the air is dead and all you can hear is the low buzzing of a fan. You drop your bag in the hall closet - it makes a thud as it hits the floor. It is quiet and you can hear your footsteps as you cross the kitchen tile. You swing open the fridge and a cold draft hits you. You pull out a small milk carton and set it on the table, then cross to the dishwasher, pull out a cup, set it on the table and fill it. You sit, take a big sip. The milk is cold and makes you shiver. Sadly, you pull yourself up and swing open the fridge again. There's a plastic container with half of yesterday's can of chicken soup in it. On the shelf below there's a foam take-out box with the remains of the beef and rice from the night before. You close the fridge door and sit back down.

Should you get take-out delivered again? Maybe pizza this time. For a special occasion. Some occasion! Why did you come to this city? You may not have liked everybody in your little town, but at least you had your family and a few good friends. And people knew one another, looked out for one another. Despite the fact that you have been in the city for a while now you haven't made one friend. And now your partner's gone, too: couldn't take it. Headed back to that little town. Or maybe couldn't take you. Guess you'll never know, since your ex won't speak to you or even email.

You pull out your phone and turn it on. No messages, not even from anyone in your family. You tug the fridge door open and pull out the beef and rice, pop it in the microwave, and stand waiting for the ping. The microwave signals it's done and you grab the food and a fork from the dishwasher, then cross into the main room. You drop yourself onto the empty love seat and punch the remote. Canned laughter bleats from the TV. It's a rerun of an old sitcom about a group of friends. You take a bite of beef and your stomach knots. Tears fill your eyes. It's your birthday. And you've never felt so lonely.

Pause for a moment and think about the story you just read. How would you feel if this really was your situation?

<u>Economic prospects: Safe condition</u>

You turn into the parking lot and park your new car, careful not to scratch it on the post. There's a spring in your step as you cross the lot and step through the sliding doors into your bank. You walk up to the Loans and Mortgage desk and pull the original papers out of your bag. The lady behind the desk looks puzzled, but she smiles at you and her eyes sparkle through her glasses.
"Can I help you?" she asks. You realize you're grinning as you pass the mortgage papers to her. "I want to finish paying this off, please" "Oh, well, that's nice. All in one payment?"
"That's correct", you say. She smiles back at you. "Let me pull up your file." She taps away at her keyboard and studies the file for a moment. "Yes, you can do that without penalty under these terms. Did you want me to transfer it from your account?" You grin even harder and nod.

It's taken many years to get to this point, but it's still nowhere near as long as you had once expected. Now everything is happening quickly. It's not as if you haven't worked hard for it, and you'll keep working hard. Well, that's assuming you'll keep working. Maybe you could retire early? You've slowly worked your way up in the company, and you've been responsible and diligent. You're known as a good worker, and your job is secure. Financially you've always been fairly comfortable, and had enough for a bit more than the basics. But you've sometimes had to be careful with your money. Restaurants not too often, mostly short vacation trips, and you've driven the same car for quite a few years until yesterday.

But thanks to poor Uncle Bill, that's all changed. Last month the inheritance money from your great uncle came through to your account. It's enough to buy a new car, pay down the remaining fifteen years on the home mortgage and still have enough to add to the company pension and saving a good chunk of cash for the future. The money situation has been pretty good in recent years, but now, with the

mortgage taken care of, you'll own your own home. As the mortgage and loans lady stamps "Paid" across your mortgage papers, you feel fully financially secure and at ease.

Pause for a moment and think about the story you just read. How would you feel if this really was your situation?

<u>Economic prospects: Harsh condition</u>

You step off the bus and begin the one block walk to your bank that's located in the middle of an old strip mall. As you get close to the mall you walk slowly across a dusty parking lot, rehearsing once again what you'll say. You notice for the first time that the sole on your shoe is splitting off. Maybe you can glue it when you get home. You hope the bank people don't notice. You think to yourself, "How did you ever get into this situation?" You've never been rich, but now you're hitting bottom.

It started when the company went out of business - without any warning. No severance, nobody to sign the employment statement that EI—Employment Insurance Canada--wanted. After weeks of filling out forms and going from one stubborn bureaucrat to another, you finally you got them to accept the pay stubs and the news article about the company's demise as proof. You looked for work but the downturn has made jobs really scarce. Then unemployment insurance ran out and the car was repossessed. And now this.

Your heart beats rapidly as you walk into the bank. You get into the lineup for tellers, and stand there for a few minutes, sweaty hands in your pockets, shuffling along behind some guy in a suit, whose new shoes shine under the fluorescent lights. Your stomach grumbles and you think about one more lunch of Kraft dinner. You then notice there's a different counter that says "Loans and Mortgages". You feel a bit foolish for not noticing it sooner. You cross over and look towards the lady standing behind the counter, but you can't look her in the eye. You reach into your bag and pull out the mortgage papers and the threatening letters you've been getting from the bank for several months. You look at the lady smiling innocently at you. At least this isn't the angry one you had to talk with last month, who kept you waiting for an hour and sneered at you when she had to give you a one-month extension. Your stomach cramps. You're already four months overdue. Why would they give you yet another extension? They'll own the house completely in two days, and you'll be on the street. This time, it feels hopeless. You're not going to make it out of this hole. You really don't know what you're going to do.

Pause for a moment and think about the story that you just read. How would you feel if this really was your situation?

<u>Physical Safety: Safe condition</u>

As you amble along the elm-lined street, a white-haired man raking golden leaves on his lawn smiles and waves. You smile and wave back, then look up at the way the late afternoon sun makes the elm leaves glow above you. You love taking your time on the way home from work—it's a pleasant and relaxing walk and most people here are friendly or at least look completely harmless. Some of them

remember you from your childhood or at least know your family. There's hardly any traffic, and the few cars you see are going very slowly. Recently, you managed to get a job transfer back to your home town which made you happy. You don't miss the city at all.

In the city, you used to have to walk through the worst section of town at least twice a day. You looked around constantly to ensure you were safe. But all that's been behind you now. Today you are feeling safe and breathing a lot easier.

The wonderful thing about this town is the way everyone keeps an eye out for you. Not that the local police are not good at what they do. It's just that they don't have that much to do. Bob, the local cop, seems to spend most of his time at the downtown coffee shop, because there's rarely much need for his services. That's why you feel a bit silly as you come up to your house and pull out your new set of keys. The keys were brought to your office today by the local locksmith. You guess you must have brought your old city mentality with you when you asked him to change the locks and install a new deadbolt on your front door.

The new door handle and locks sparkle brightly in the afternoon sun. You open the lock for the first time. The key slips in snugly and easily. You unlock the handle lock, then the dead bolt, then walk through the door. As you close it behind you and hear a comforting click as the door closes securely behind you. You feel completely, utterly safe.

Pause for a moment and think about the story you just read. How would you feel if this really was your situation?

Harsh Environment: Physical Safety

It's after midnight and you are on the last bus of the night. You reach up and pull the cord to signal your stop - the bell rings. You ride this bus in daylight every day but haven't had to come home so late before. You stand, the brakes squeal and you are thrown forward as the bus pulls up to your stop. You're hesitant to get off, but you'll have a lot further to walk if you don't move quickly. As you step onto the hard pavement you pause, thinking once again about the body that was found in the alley behind the bus stop last month.

The bus hisses and clanks, pulling away in a fog of diesel fumes. You ask yourself, "what got into your head a few years ago when you left the safety of your little town for this dark and dangerous city?"

You take a deep breath and peer into the dark alley - nothing but darkness. You survey the rust-riddled parked cars along the road. Hopefully, no one is lurking behind them. Then you see a guy sitting behind the wheel across the street, his face shadowed. A dog barks and snarls somewhere nearby. You move quickly along the sidewalk, past a row of pawn shops and cash stores, their smudged windows shuttered with steel grating.

You hear the rumble of a big car behind you. It drifts slowly along. It feels like it is following you. You glance back quickly. It's the car with the shadowy-faced driver you saw on the other side of the

street. You see his face this time—a sharp face, smiling weirdly, watching you. You pick up your pace and are breathing hard as you cover the last block. He's still there, the car crawling along beside you like a wolf following you at the edge of a forest. You turn off on your side street and he drifts by.

The lights along this street are out for some reason. It's dark but you're breathing a little easier to see that the car has missed the turn and drifted on past. Then, as you make the corner, you see light swing across the apartments on your left. You look behind and see headlights approaching. They blind you at first. Then, as the car creeps closer and slows down, you see it's the same car. As it gets closer to you, your heart races and your whole body tightens like a vice.

Pause for a moment and think about the story you just read. How would you feel if this really was your situation?
